# Partial Netrin-1 Deficiency Aggravates Acute Kidney Injury

**DOI:** 10.1371/journal.pone.0014812

**Published:** 2011-05-19

**Authors:** Almut Grenz, Julee H. Dalton, Jessica D. Bauerle, Alexander Badulak, Douglas Ridyard, Aneta Gandjeva, Carol M. Aherne, Kelley S. Brodsky, Jae-Hwan Kim, Rubin M. Tuder, Holger K. Eltzschig

**Affiliations:** 1 Mucosal Inflammation Program, Department of Anesthesiology, University of Colorado Denver, Anschutz Medical Campus, Aurora, Colorado, United States of America; 2 Division of Pulmonary Sciences and Critical Care Medicine, University of Colorado Denver, Anschutz Medical Campus, Aurora, Colorado, United States of America; 3 Department of Anesthesiology, Korea University College of Medicine, Seoul, Republic of Korea; Institut Pasteur, France

## Abstract

The netrin family of secreted proteins provides migrational cues in the developing central nervous system. Recently, netrins have also been shown to regulate diverse processes beyond their functions in the brain, incluing the ochrestration of inflammatory events. Particularly netrin-1 has been implicated in dampening hypoxia-induced inflammation. Here, we hypothesized an anti-inflammatory role of endogenous netrin-1 in acute kidney injury (AKI). As homozygous deletion of netrin-1 is lethal, we studied mice with partial netrin-1 deletion (*Ntn-1^+/−^* mice) as a genetic model. In fact, *Ntn-1^+/−^* mice showed attenuated Ntn-1 levels at baseline and following ischemic AKI. Functional studies of AKI induced by 30 min of renal ischemia and reperfusion revealed enhanced kidney dysfunction in *Ntn-1^+/−^* mice as assessed by measurements of glomerular filtration, urine flow rate, urine electrolytes, serum creatinine and creatinine clearance. Consistent with these findings, histological studies indicated a more severe degree kidney injury. Similarly, elevations of renal and systemic inflammatory markers were enhanced in mice with partial netrin-1 deficiency. Finally, treatment of *Ntn-1^+/−^* mice with exogenous netrin-1 restored a normal phenotype during AKI. Taking together, these studies implicate endogenous netrin-1 in attenuating renal inflammation during AKI.

## Introduction

Acute kidney injury (AKI) is defined a decrease in the glomerular filtration rate (GFR), occurring over a period of minutes to days. AKI is frequently caused by renal ischemia, and represents an important cause of morbidity and mortality of hospitalized patients [1], [2], [3]. A recent study revealed that only a mild increase in the serum creatinine level (0.3 mg/dl) is associated with a 70% greater risk of death than in patients without any increase [2], [3]. Along these lines, surgical procedures requiring cross-clamping of the aorta and renal vessels are associated with a rate of AKI of up to 30% [4]. Similarly, acute renal failure after cardiac surgery occurs in up to 10% of patients under normal circumstances and is associated with dramatic increases in mortality [5]. Moreover, patients with sepsis frequently go on to develop AKI and the combination of moderate sepsis and AKI is associated with a 70% rate of mortality. Therapeutic approaches are very limited and the majority of interventional trials in AKI have failed in humans [6]. Therefore, additional therapeutic modalities to prevent or treat AKI presently represent an area of intense investigation [7].

Named after the Sanskrit word netr, which means ‘one who guides’, the netrin family of secreted proteins provides migrational cues in the developing central nervous system. More recently, netrins have been shown to regulate diverse processes (such as cell adhesion, motility, proliferation, differentiation and, ultimately, cell survival) in a number of non-neuronal tissues [8]. The ability of the guidance molecule netrin-1 (Ntn-1) to repulse or abolish attraction of neuronal cells makes it an attractive candidate for the regulation of inflammatory cell migration. In fact, previous studies have shown that Ntn-1 is involved in the orchestration of inflammatory responses *in vitro* or *in vivo* [9], [10]. Particularly, netrin-1 has been implicated in regulating inflammatory events during conditions of tissue hypoxia [9]. Given that mucosal surfaces are particularly prone to hypoxia-elicited inflammation, a recent study sought to determine the function of netrin-1 in hypoxia-induced inflammation [9]. The authors observed hypoxia-inducible factor 1alpha (HIF-1α)-dependent induction of expression of the gene encoding *Ntn1* in hypoxic epithelia. Neutrophil transepithelial migration studies showed that by engaging A2B adenosine receptor (A2BAR) on neutrophils, netrin-1 attenuated neutrophil transmigration. Another study demonstrated that endothelial netrin-1 interacts with inflammatory cells and is capable of attenuating inflammation by potent inhibition of myeloid cell migration [10]. Taken together, these studies indicate that netrin-1 attenuates organ injury by hypoxia or inflammation [9].

Recent studies in the kidney showed that exogenous netrin-1 treatment in mice or renal ischemia in mice overexpressing renal netrin-1 attenuated kidney injury following ischemia [11], [12]. The same authors showed that UNC5B receptors on leucocytes attenuate kidney injury due to ischemia [13]. Furthermore they showed that urinary netrin-1 excretion is increased in patients following acute renal failure compared to urine from healthy controls suggesting netrin-1 as an early biomarker for acute renal failure [14]. However, studies in gene-targeted mice for netrin-1 have not been performed.

Due to the fact that AKI is characterized by an acute inflammatory event in the context of tissue hypoxia, we hypothesized a role of endogenous netrin-1 in dampening ischemia-driven inflammation and kidney dysfunction during AKI. We were particularly interested in identifying the role of endogenous netrin-1 in this response – as the adult kidney was previously shown to be the single organ with the highest expression of netrin-1 (even higher than the brain) [10]. Based on previous studies showing that netrin-1 is induced during conditions of limited oxygen availability (hypoxia), and dampens hypoxia-induced inflammation [9], we hypothesized a role for endogenous netrin-1 in dampening renal failure after AKI. In fact, studies of in gene-targeted mice for netrin-1 confirmed our hypothesis and indicate a protective role of endogenous netrin-1 in AKI.

## Material and Methods

### *In vivo* model of AKI

The animal protocol was approved by the Institutional Animal Care and Use Committee of the University of Colorado Denver, and is in accordance with the National Institutes of Health guidelines for use of live animals. Previously described *Ntn-1^+/−^* mice or *Ntn-1^+/+^* mice littermate controls matched in age, gender, and weight were used [15].

Mice were anaesthetized using 50 mg/kg i.p. pentobarbital and underwent right nephrectomy followed by left renal artery ischemia for 30 minutes by using a hanging weight system, as previously described [16], [17], [18], [19]. Plasma creatinine and urine creatinine and potassium were measured 24 hours following renal ischemia by the hospital laboratory and kidneys were harvested and stored at −80°C until further analysis. Inulin clearance was measured 60 minutes following renal ischemia as described previously [17]. Briefly, the right jugular vein was cannulated for continuous infusion. Next, 0.75% FITC-inulin was added to the infusion for determination of glomerular filtration rate (GFR). Blood samples were taken via retroorbital puncture. A catheter was placed in the urinary bladder for timed urine collection. Three urine collection periods were performed with blood collection in the middle of the period. FITC-inulin concentrations in plasma and urine samples were measured.

### Cell culture and hypoxia exposure

Human renal epithelial cells (HK-2) were exposed to normobaric hypoxia (1% O_2_, 99%N_2_) in a hypoxic chamber (hypoxic glove box, COY Laboratory Products INC. Michigan, USA) over indicated time periods.

### Transcriptional Studies

We used real-time RT-PCR (iCycler; Bio-Rad Laboratories Inc.) to examine Ntn-1, IL-6, TNF-α and IL-10 expression in renal tissue as previously described [9], [13]. Primer sets (sense sequence, antisense sequence, and transcript size, respectively) for the following genes were: netrin-1 (5′- CTCACAGCAATGTCAACAGC -3′, 5′- GCAGGAAGCAGTCACAGAAT -3′, 191 bp); IL6 (5′ - CGG AGA GGA GAC TTC ACA GA -3′, 5′ - CCA GTT TGG TAG CAT CCA TC -3′, 218 bp); TNF-α (5′- CCCACTCTGACCCCTTTACT -3′, 5′- TTTGAGTCCTTGATGGTGGT -3′, 201 bp); IL-10 (5′-CCCAAGTAACCCTTAAAGTCCTTGC, 5′-ATGCTGCCTGCTCTTACTGACTG-3′, 200 bp). Murine ß-actin mRNA (5′- CTAGGCACCAGGGTGTGAT -3′, 5′- TGCCAGATCTTCTTCATGTC -3′) was amplified in identical reactions to control for the amount of starting template.

### Renal histology

Kidneys were excised and harvested 24 hours following 30 minutes of ischemia. Paraffin-embedded sections (3 µm) were stained with hematoxylin and eosin or rat anti-mouse neutrophil MCA771G antibody (Abd Serotec, NC, USA) to assess neutrophil accumulation in the kidneys [17].

### Human and mouse protein analysis

Renal tissues and renal epithelial cells (HK-2 cells) were blotted using polyclonal rabbit anti-netrin-1 (Calbiochem Laboratories) and polyclonal goat anti-netrin-1 (Santa Cruz), respectively [9].

### Immunohistochemistry

Immunohistochemistry for *NTN-1* on 3-µm-thick kidney sections was performed using formalin-fixed, paraffin-embedded tissue and sections that were stained with polyclonal rabbit anti-mouse netrin-1 (Calbiochem Laboratories) [9].

### Netrin-1 ELISA

Netrin-1 in renal tissue and urine was measured using a commercially available ELISA kit (Hoelzel Diagnostika, Germany).

### Statistic analysis

Data are presented as mean ± SD from four to six animals per condition. We performed statistical analysis using the Student *t* test (two sided, <0.05). Renal injury scores were analyzed with the Kruskal-Wallis rank test and are given as median ± range. A value of *p*<0.05 was considered statistically significant.

## Results

### Renal netrin-1 expression in Ntn-1^+/−^ mice

**Figure 1:**
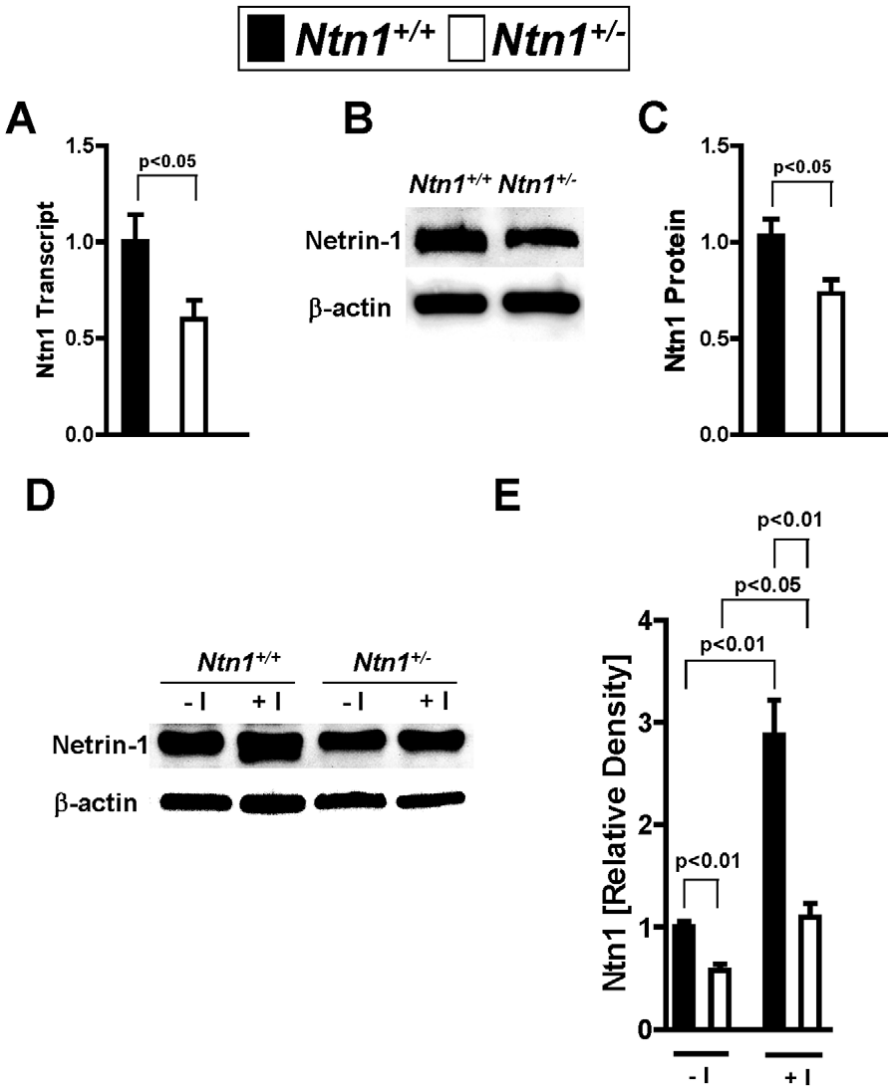
Renal netrin-1 expression in wild-type mice (*Ntn1^+/+^*) or mice with partial netrin-1 deficiency (*Ntn1^+/−^*). (A) Real-time RT-PCR analysis of the expression of *Ntn1* mRNA in the kidneys of *Ntn1^+/+^* and *Ntn1^+/−^* mice, presented relative to β-actin. Data are representative of four mice in each group (mean ± SD). (B,C) Expression of netrin-1 protein in *Ntn1^+/+^* or *Ntn1^+/−^* mice. (D,E) Expression of netrin-1 protein in kidneys of *Ntn1^+/+^* or *Ntn1^+/−^* mice following 30 minutes of ischemia and 2 hours reperfusion (+I) compared to control kidneys without ischemia (−I). Each blot was re-probed for β-actin to control for loading. One representative of three blots is displayed.

**Figure 2:**
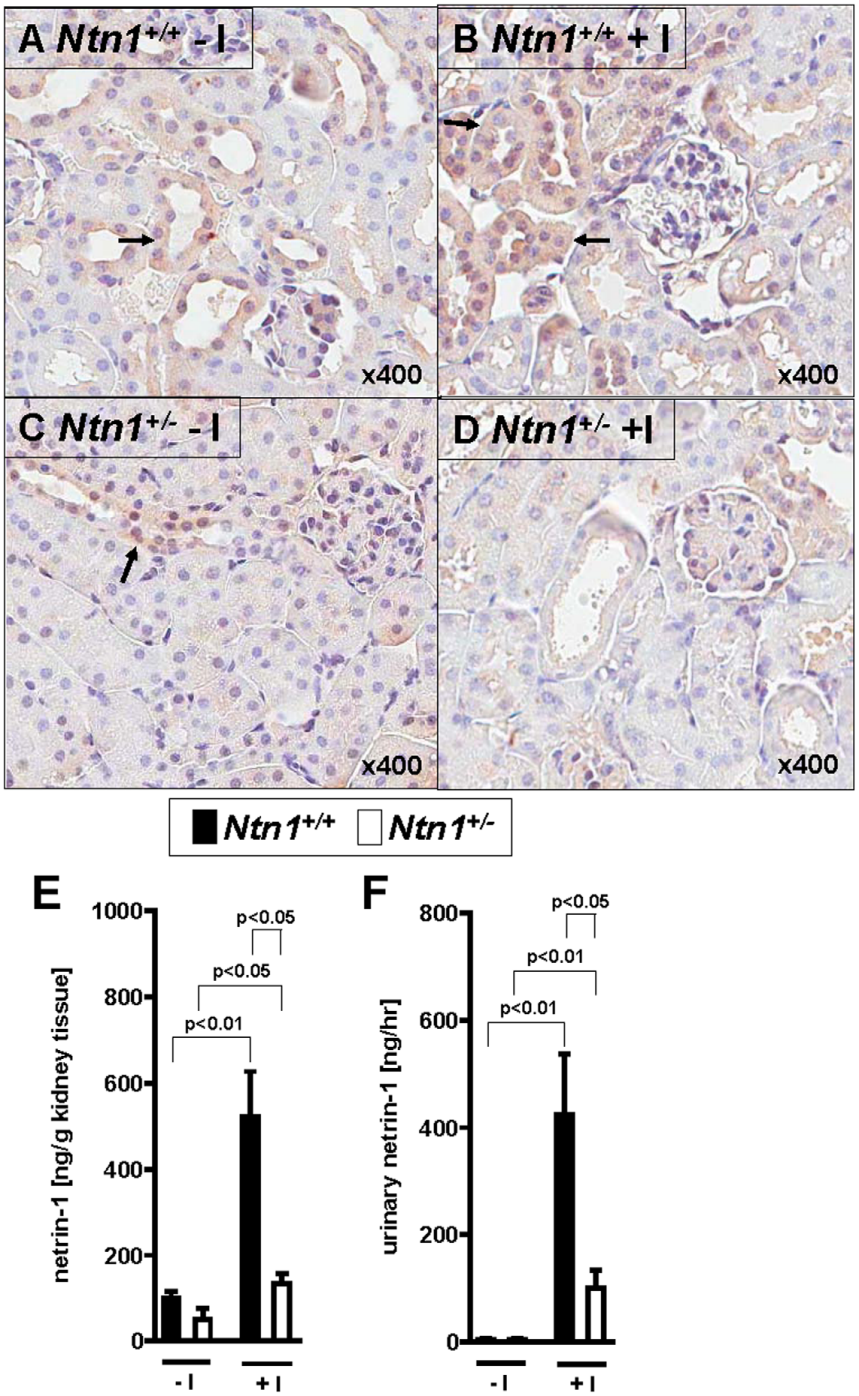
Immunohistochemical localization of renal netrin-1 and netrin-1 tissue content and urine concentration following ischemia *in vivo*. Immunohistolochemical staining for netrin-1 in kidneys of *Ntn1^+/−^* mice or their respective age-, weight-, and gender-matched littermate controls (*Ntn1^+/+^*) following 30 minutes ischemia and 2 hours reperfusion. (A) Netrin-1 protein is mainly expressed in tubule cells of *Ntn1^+/+^* mice under basal conditions without ischemia (−I) and (B) is increased following ischemia (+I). (C,D) This increase of netrin-1 expression following ischemia is attenuated in *Ntn1^+/−^* mice. Arrows indicate tubules with netrin-1 expression. (magnification 400×). (E) Renal and (F) urine netrin-1 content were assessed by ELISA (mean ± SD; n = 6–8).

**Figure 3:**
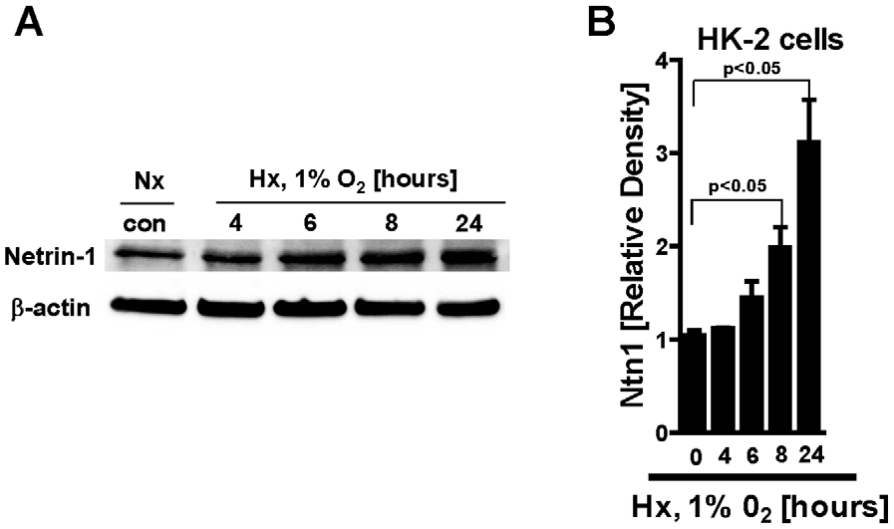
*In vitro* expression of netrin-1 in HK-2 cells. (A) Expression of netrin-1 in human renal epithelial cells (HK-2 cells) following exposure to hypoxia (1% O_2_) for indicated time periods. One representative blot of three is displayed. (B) Quantification or netrin-1 protein in HK-2 cells relative to β-actin.

To study the role of endogenous netrin-1 in AKI, we utilized previously described mice with genetic deletion of netrin-1 [9], [15]. Homozygote mice gene-targeted for netrin-1 are not viable, and die shortly after birth [15]. Therefore, we examined mice with partial netrin-deficiency (*Ntn-1^+/−^* mice) [9]. Initial characterization of renal netrin-1 expression in *Ntn-1^+/−^* mice revealed significant reduction of netrin-1 transcript and protein levels at baseline (Figure 1A–C). As next step, we induced ischemic AKI in wild-type mice (30 min of renal ischemia followed by 2 h of reperfusion, Figure 1 D, E) and observed robust increases of renal netrin-1 levels. In contrast, renal netrin-1 levels in *Ntn-1^+/−^* mice exposed to AKI remained approximately at wild-type baseline levels. Immunohistochemistry for renal netrin-1 indicated dominant netrin-1 expression in proximal tubular cells, in conjunction with increased netrin-1 levels following renal ischemia. In contrast, renal netrin-1 staining at baseline or following renal ischemia was attenuated in *Ntn-1^+/−^* mice. To further define netrin-1 expression we measured netrin-1 in renal tissue, urine and serum via ELISA. Interestingly we could show a tremendous increase of netrin-1 in renal tissue and urine following ischemia in wild-type control mice compared to *Ntn-1^+/−^* mice, whereas serum concentrations were not detectable assuming that netrin-1 expression occurs mainly in renal epithelial cells (Figure 2E, F). Similarly, renal epithelial cells (HK-2 cells) showed robust expression of netrin-1 in conjunction with netrin-1 induction following exposure to ambient hypoxia (1% oxygen over 0–24 h, Figure 3A and B). Taken together, these studies indicate robust netrin-1 expression predominantly in renal epithelia, and suggest that mice with partial netrin-1 deficiency can be used as a model to study endogenous netrin-1 during ischemia-induced AKI.

### AKI is aggravated in Ntn-1^+/−^ mice

**Figure 4:**
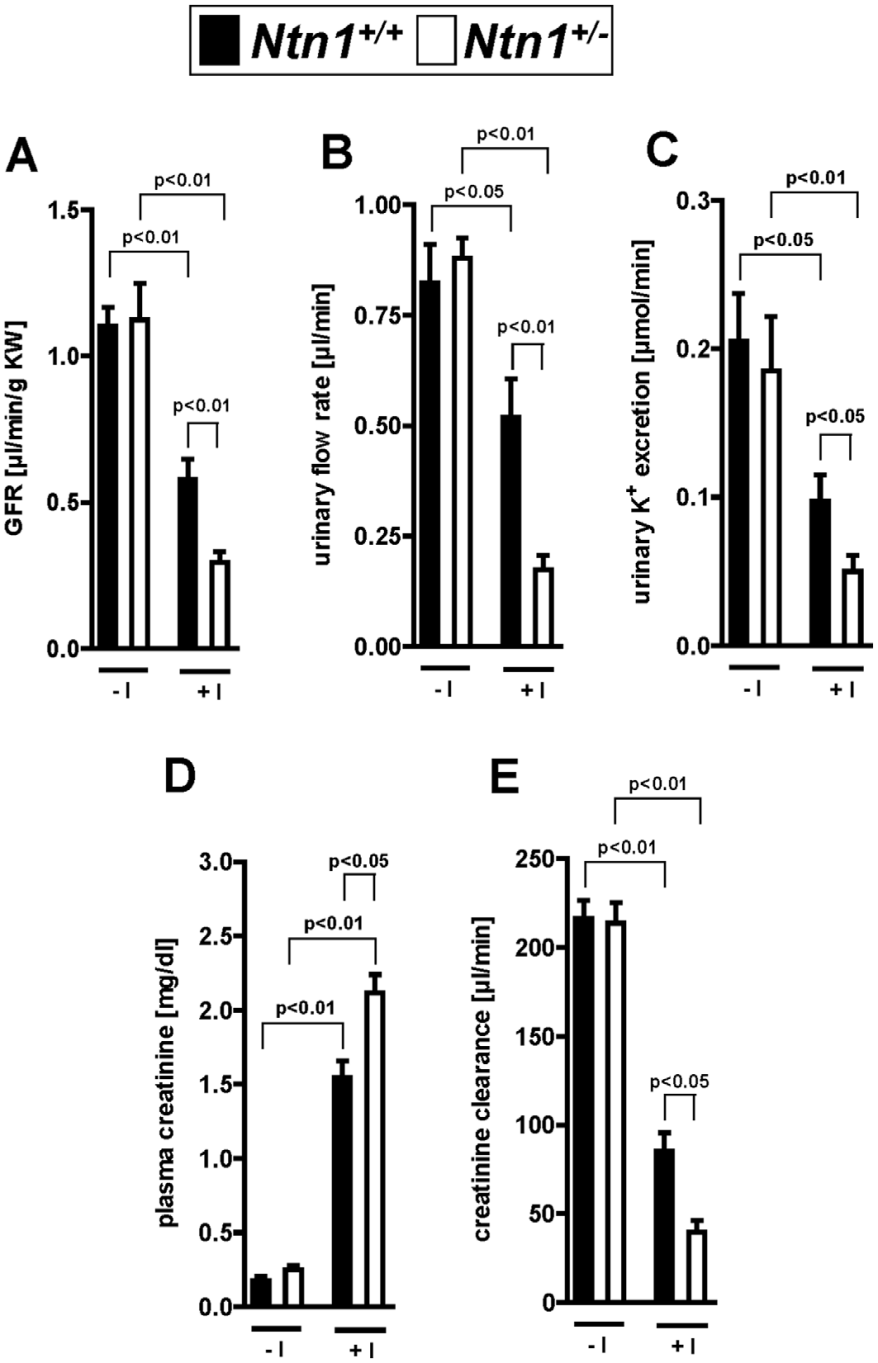
Renal function in mice with partial deficiency for netrin-1 (*Ntn1^+/−^*) exposed to ischemic AKI. Previously characterized *Ntn1^+/−^* mice or their respective age-, weight-, and gender-matched littermate controls (*Ntn1^+/+^*) underwent right nephrectomy and were exposed subsequenctly to AKI induced by 30 minutes of left renal artery ischemia. (A) Glomerular filtration rates (as measured by FITC-inulin clearance) were obtained after 1 hour of reperfusion. (B) Urinary flow rate, (C) urinary potassium excretion, (D) serum creatinine and (E) creatinine clearance were obtained 24 hours following reperfusion. Data are representative of six to eight independent experiments for each experimental condition (mean ± SD).

**Figure 5:**
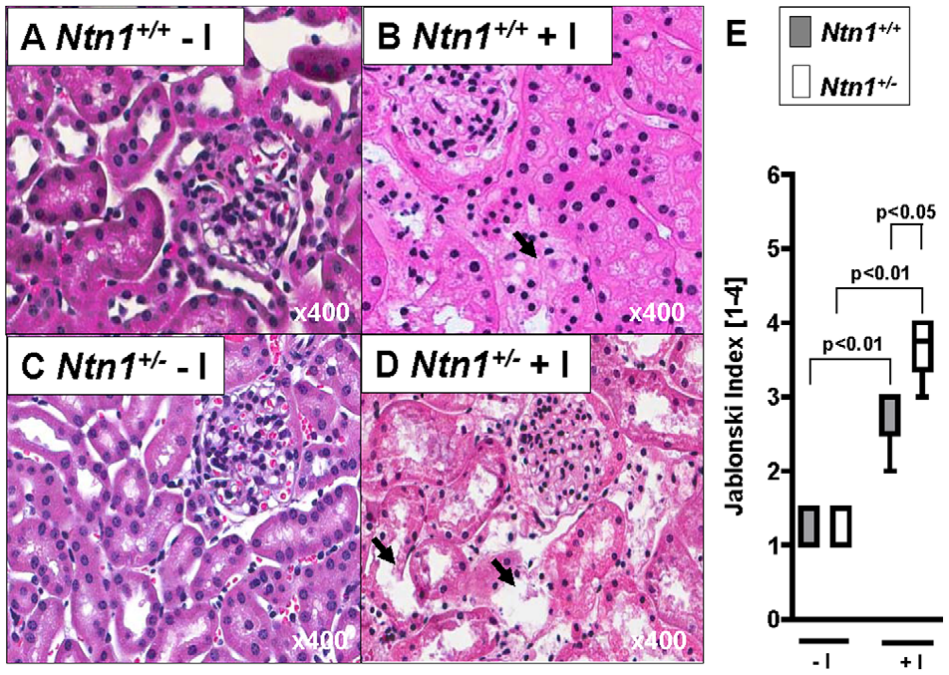
Histological tissue insure induced by AKI in mice with partial netrin-1 deficiency (*Ntn1^+/−^*) or control mice (*Ntn1^+/+^*). Renal histology in *Ntn1^+/−^* mice exposed to renal ischemia or age-, weight-, and gender-matched littermate controls (*Ntn1^+/+^*) were subjected to 30 minutes of left renal artery ischemia. Renal histology was obtained after 24 hours of reperfusion. (A–D) Representative H&E staining (400×). Arrow marks destructed tubules. (E) Quantification of histological tissue damage assessed by Jablonski index.

After having characterized renal netrin-1 expression at baseline or following AKI, we next pursued functional studies of AKI in *Ntn-1^+/−^* mice. For this purpose, we utilized a previously described model of ischemia induced AKI where isolated renal artery occlusion is achieved via a hanging weight system, thereby minimizing surgical trauma [16], [17], [18], [19]. In short, we performed a unilateral nephrectomy, followed by selective left renal artery occlusion via a hanging weight-system in the remaining kidney to induce AKI [16]. Following 30 min of renal ischemia and 1 hour of reperfusion, we measured glomerular filtration rate by infusion of FITC-labeled inulin via a jugular vein infusion catheter. These studies revealed a significantly enhanced decrease in renal GFR following AKI induction in *Ntn-1^+/−^* mice as compared to littermate controls matched in age, gender and weight (Figure 4A). Similarly, measurements of urinary flow rate, potassium excretion, serum creatinine and creatinine clearance measured 24 hours following renal ischemia, indicate a more severe degree of AKI in mice with partial netrin-1 deficiency (Figure 4B–E). Moreover, studies of renal histology demonstrate more severe acute tubular necrosis—obvious from the loss of tubular cell nuclei in the cortex and outer medulla with destruction of the proximal tubular brush border. In addition, hyaline cast formation, intraluminal necrotic cellular debris, and casts containing brush border blebs were more predominant in *Ntn-1^+/−^* mice exposed to renal ischemia (Figure 5A–D). This was confirmed utilizing a histologic score for the severity of AKI (Figure 5E). Together, these studies indicate that ischemia-induced AKI is more severe in *Ntn-1^+/−^* mice.

### AKI induced renal inflammation is enhanced following partial Ntn-1^+/−^ deficiency

**Figure 6:**
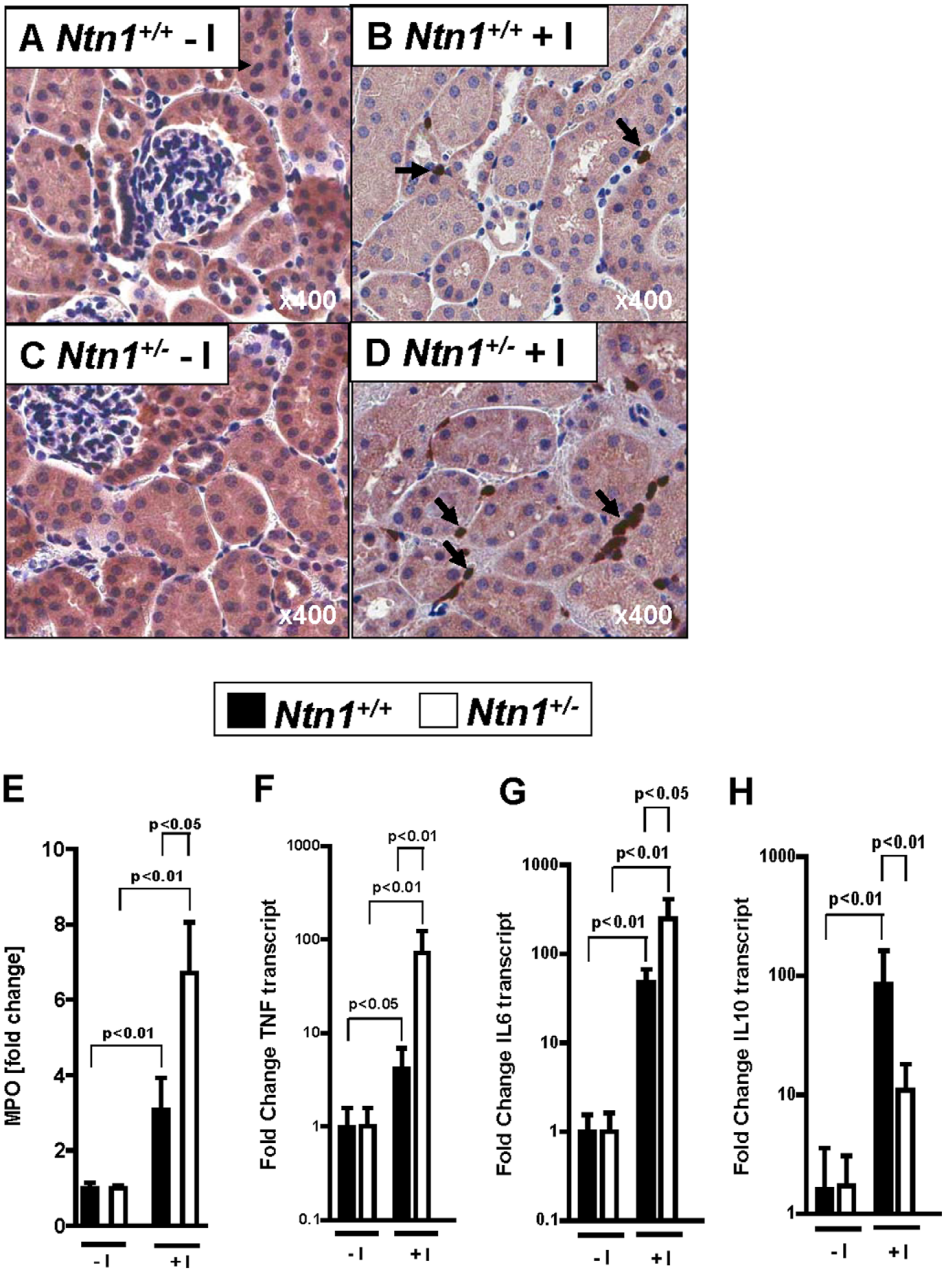
Renal inflammatory changes in *Ntn1^+/−^* mice following ischemia. *Ntn1^+/−^* mice and their respective age-, weight-, and gender-matched littermate controls (*Ntn1^+/+^*) were subjected to 30 minutes of left renal artery ischemia. (A–D) Neutrophil staining. Arrows indicate neutrophils (magnification 400×). (E) Quantification of neutrophil tissue accumulation by measurement of myeloperoxidase (MPO). (F) TNF-α and (G) interleukin-6 (IL-6) and (H) interleukin-10 (IL-10) were assessed by real-time RT-PCR from renal tissues. Data were calculated relative to ß-actin and are expressed as fold change compared to sham-operated animals without ischemia (−I). Data are representative of four to six independent experiments for each experimental condition (mean ± SD).

Based on previous studies indicating that netrin-1 signaling dampens acute inflammatory events induced by hypoxia [9], we went on to assess the role of endogenous netrin-1 in AKI-induced renal inflammation. Here, histological staining, or measurements of renal myeloperoxidase indicate that neutrophil accumulation following AKI is enhanced in *Ntn-1^+/−^* mice (Figure 6A–E). Moreover, AKI-induced elevations of renal inflammatory markers including TNF-α and IL-6 were enhanced whereas the anti-inflammatory cytokine IL -10 was reduced in mice with partial netrin-1 deficiency (Figure 6F–H). Together, these studies suggest that endogenous netrin-1 signaling represents an endogenous feedback loop to dampen AKI-induced inflammation of the kidneys.

### Reconstitution of Ntn-1^+/−^ mice during AKI

**Figure 7:**
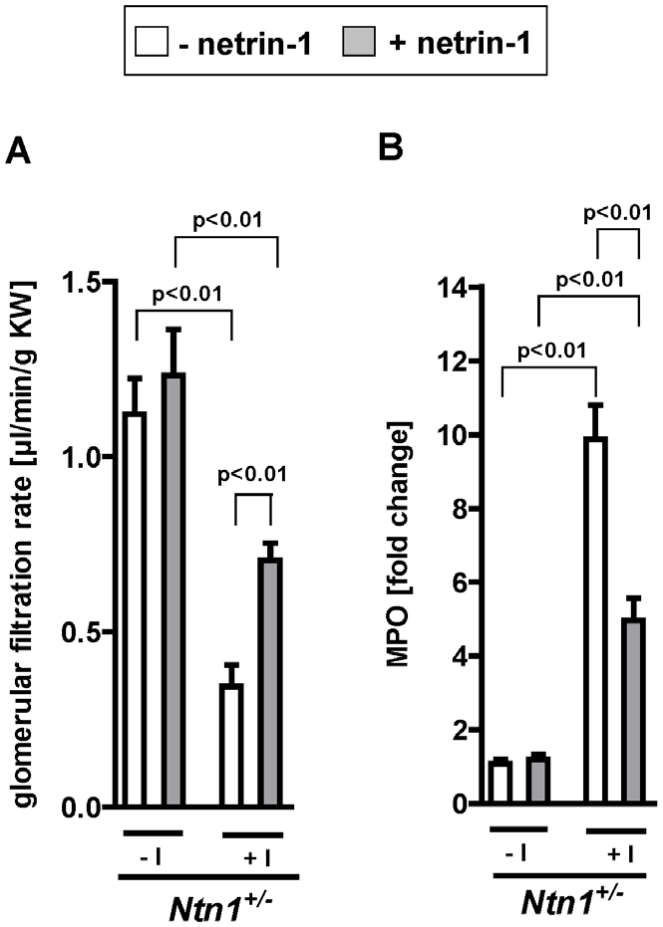
Reconstitution of *Ntn1^+/−^* mice with exogenous netrin-1. Renal function and inflammation in mice with partial deficiency for netrin-1 (*Ntn1^+/−^*) treated with exogenous netrin (5 µg/mouse I.V.) or vehicle prior to 30 minutes of renal ischemia. (A) Glomerular filtration rate (as measured by FITC-inulin clearance) was measured after 1 hour of reperfusion. (B) Quantification of neutrophil tissue accumulation by measurement of myeloperoxidase (MPO) (mean ± SD; n = 6–8).

As proof of principle for the assertion that netrin-1 plays an important role in the regulation of renal injury and kidney inflammation during AKI, we reconstituted *Ntn-1^+/−^* mice with exogenous netrin-1 (5 µg/mouse I.V. 30 min prior to induction of AKI) [9]. We have chosen this netrin-1 dose based on previous studies testing different netrin-1 doses (0.5, 1, 5, 10 µg/mouse I.V.) showing the strongest renal protection from ischemia injury with 5 µg/mouse. In fact, these studies revealed that reconstitution with exogenous netrin-1 restored a wild-type phenotype for AKI-induced changes of GFR or renal inflammation following AKI in *Ntn-1^+/−^* mice (Figure 7A and B). Taken together, these studies provide strong evidence that netrin-1 signaling is a critical control point for kidney inflammation and tissue injury following AKI.

## Discussion

Tissue hypoxia during AKI results in severe kidney inflammation, including inflammatory cell accumulation, cytokine release, and inflammation-associated organ dysfunction. Based on recent studies suggesting a role of the neuronal guidance molecule netrin-1 in dampening hypoxia-elicited inflammation [9], we examined the role of netrin-1 in AKI. Utilizing mice with partial netrin-1 deficiency (*Ntn-1^+/−^* mice) we found that these mice are more prone to AKI-induced kidney dysfunction and renal inflammation. Moreover, reconstitution of *Ntn-1^+/−^* mice with exogenous netrin-1 retreatment resuscitated their phenotype. Taken together, these studies provide the first genetic *in vivo* evidence for a critical role of endogenous netrin-1 in attenuating AKI-driven renal dysfunction and inflammation.

At present, the signaling pathways involving renal protection through netrin-1 remain unclear. Previous studies have indicated that endogenous netrin-1 is released into the urine, and can serve as an early biomarker of AKI [14], [20]. Other studies suggest that netrin-1 signaling via activation of the UNC5B receptor protects the kidneys from ischemia [11], [21]. Moreover, a very elegant study utilizing a genetic model of netrin-1 overexpression demonstrates that netrin-1 signaling protects the kidneys from ischemia reperfusion injury by suppressing tubular epithelial apoptosis [12]. Finally, another study suggests that anti-inflammatory signaling events of netrin-1 involve the A2B adenosine receptor (A2BAR), particularly during conditions of hypoxia-elicited inflammation [9]. While the mechanisms of how netrin-1 interacts with the A2BAR remain unclear, this study demonstrates that netrin-1 signaling events enhance adenosine-dependent tissue protection from hypoxia [9], [17], [22], [23], [24], [25]. This assumption would be consistent with other studies on the role of the A2BAR in myocardial ischemia [23], [25], vascular leakage [26], [27], intestinal inflammation [24], [28], [29], or acute lung injury [22], [30], where hypoxia-elicited induction of the A2BAR [31] attenuates organ inflammation and dysfunction. In fact, hypoxia has been shown to drive a coordinated adenosine response of different tissues [32], [33], [34], [35], including increased adenosine production [36], [37], [38], [39], [40], [41], [42], [43], [44], induction of the A2BAR [45], attenuated adenosine uptake [46], [47], [48] and metabolism [49], thereby enhancing anti-inflammatory and protective tissue responses during acute hypoxia [50], [51], [52]. In fact, extracellular adenosine signaling has been strongly implicated as a therapeutic in different models of kidney injury, including AKI [17], [18], [19], [23], [53], [54], [55].

Taken together, the present results identify endogenous netrin-1 as an endogenous anti-inflammatory during AKI. Future studies will have to determine the exact contribution of different tissues, e.g. by utilizing tissue-specific approaches of netrin-1 deletion, or its receptor in the kidneys. Moreover, a more mechanistic understanding of netrin-1 signaling events would set the stage for targeting netrin-1 in the treatment of patients suffering from AKI, e.g. by designing specific netrin-1 mimetic peptides. Finally, future challenges require to address the consequences of acute versus a more chronic activation of netrin-1-dependent signaling pathways. For instance, previous studies have implicated chronic activation of adenosine signaling pathways in promoting a chronic form of disease and tissue fibrosis [56], [57], [58], [59], whereas adenosine signaling events in an acute setting dampen inflammatory responses and contribute to the resolution of injury [24], [29], [30], [35], [39], [46], [51], [60], [61].
